# Film dance creation practice supported by Cyber Physical System

**DOI:** 10.1371/journal.pone.0284478

**Published:** 2023-04-28

**Authors:** Zhiqun Lin, Yulin Zhang

**Affiliations:** 1 College of Music, Capital Normal University, Beijing, 100048, China; 2 School of Education, Beijing Dance Academy, Beijing, 100081, China; Nottingham Trent University School of Science and Technology, UNITED KINGDOM

## Abstract

The traditional eight arts include film and dance. Dance is often included in the creation of films. With the progress of the times, dance has shown unprecedented vitality in film. This also puts forward higher requirements for shooting technology in film creation. To solve the contradiction between high performance of equipment and energy sensing, a new energy aware scheduling framework is proposed based on Cyber Physical System, which utilizes the balance between performance and energy consumption optimization, and uses allocation technology and list scheduling to ensure energy constraints. Besides, a highly energy-efficient and stable scheduling algorithm for film creation is constructed. The algorithm problems are mainly divided into functional safety requirements, verification problems, and energy consumption optimization problems under functional safety. The experimental results show that the system can obtain better schedulability at a lower time complexity and reasonably reflect the dynamic and static energy constraints ratio. The basic framework system based on dynamic step size also achieves better time performance than other step sizes. According to the experiment’s findings, the energy consumption of all scheduling components is relatively low and can be maintained within a specific range. The research and analysis of this study can provide a theoretical reference for the equipment algorithm of film dance creation practice, promote interaction with technical practice, and assist in promoting the development process of film dance creation.

## Introduction

The most memorable achievements of the transformation and evolution of modern human civilization are found in works of art [1]. Since modern times, the emergence of film art can more comprehensively and vividly reflect the rich cultural connotation of the times and the complex and changeable subjective spiritual world. Therefore, to avoid long-term aesthetic fatigue, film artists will use a variety of forms of expression to expand their creations. However, the combination of dance choreography, lens selection, on-site videography, and post-editing in film dance is complex, which will test the ability of equipment and filmmakers more than traditional pure film [2]. Previously, embedded systems were the core of various digital products for filmmaking [3]. The current development of information technology (IT) has led to its continuous strengthening of contact with the outside world, transforming it into a larger and more open Cyber Physical System (CPS) and the Internet of Things (IoT) [4]. CPS enhances the dynamic interaction between information systems and physical systems [5]. The IoT can improve the information interaction between smart terminal devices. However, from the perspective of the system’s energy consumption, high energy consumption is the biggest problem that needs to be solved. On the one hand, in the case of multi-threading, much heat can be generated, and temperature surge. As a result, the life of hardware equipment is shortened, resulting in permanent failure and severe economic losses caused by data loss. On the other hand, the electromagnetic radiated energy generated by high energy consumption can cause electromagnetic interference problems. Thus, in some cases, peripheral equipment fails and malfunctions, causing unnecessary safety problems.

Based on the above background, aiming at the contradiction between equipment high performance and energy awareness, this study studies the film creation scheduling algorithm with high stability and high efficiency. Firstly, the CPS is introduced. Secondly, the safety requirements verification and energy optimization problems under functional safety conditions are modeled and analyzed. A new energy aware scheduling (EAS) framework is innovatively proposed for energy constraints. Finally, the schedulability and time performance are experimentally analyzed. The study is divided into the following parts. Section 1 is the introduction, which introduces the background, purpose, and significance of this study; Section 2 is related work, through the review of other related studies, summarizes the deficiencies in the current research, and leads to the main problems and innovation points of this study; Section 3 is materials and methods. The relevant theories of CPS are expounded, the energy consumption of the algorithm is optimized, and a new EAS framework is proposed to ensure energy constraint. Section 4 is the results and discussion. The results of the study are presented and explored in sections. The conclusions are summarized and compared with other similar studies to compare the similarities and differences between this study and other studies. Section 5 is the conclusion, which summarizes the main contributions and conclusions, and points out the shortcomings of the study and the prospects for the future.

## Related works

In recent years, low-energy parallel scheduling algorithms have also become the main research point. Maghsoud et al. (2021) considered the energy-delay product as a trade-off between energy savings and performance improvement. They proposed a technique for performing work-stealing scheduling in the operating system kernel without modifying user-space programs. The proposed scheduling used a predictive model to determine the optimal number of active cores and clock frequency of the processor as the optimal configuration for any running program at runtime to achieve the minimum energy-delay product value [6]. Lu et al. (2022) put forward a new task-scheduling algorithm called energy-aware dual-adaptive particle swarm optimization (PSO). It was an improved PSO for achieving energy efficiency and minimal task execution time in edge computing environments [7]. Ahmad et al. (2021) proposed an energy-efficient workflow scheduling algorithm. It reduced energy consumption using a fair pre-allocation of available budgets, which could be reduced within customer-specified budget constraints. Savings in energy consumption by including energy and cost factors enabled a fair distribution of the available budget for unscheduled tasks in the workflow application [8]. Huang et al. (2021) investigated scheduling methods that were independent or weakly dependent on dynamic voltage and frequency scaling to achieve task requirements in heterogeneous computing systems that must meet execution deadlines and minimize energy consumption. This method was used for parallel real-time applications with hard terms running on heterogeneous computing systems [9]. Hosseini and Noorian (2022) scheduled scientific workflows on hybrid cloud architectures that included private and public clouds. A scientific workflow scheduling problem running on a hybrid cloud architecture was expressed as a dual-objective optimization problem to minimize the duration and monetary costs. Besides, a hybrid bi-objective optimization algorithm based on simulated annealing and task replication algorithms was proposed [10].

The above study is either from the operating system kernel, from the energy and cost factors, or from the perspective of dynamic voltage and frequency, to reduce energy consumption and improve performance. These studies focused on balancing energy consumption and performance by building different systems, but did not ensure energy constraints through the EAS framework. As such, based on the CPS, the safety requirement verification problem and energy consumption optimization problem under functional safety are studied and modeled, and a new energy constraint evolution algorithm framework is innovatively put forward.

## Materials and methods

### CPS

**Fig 1:**
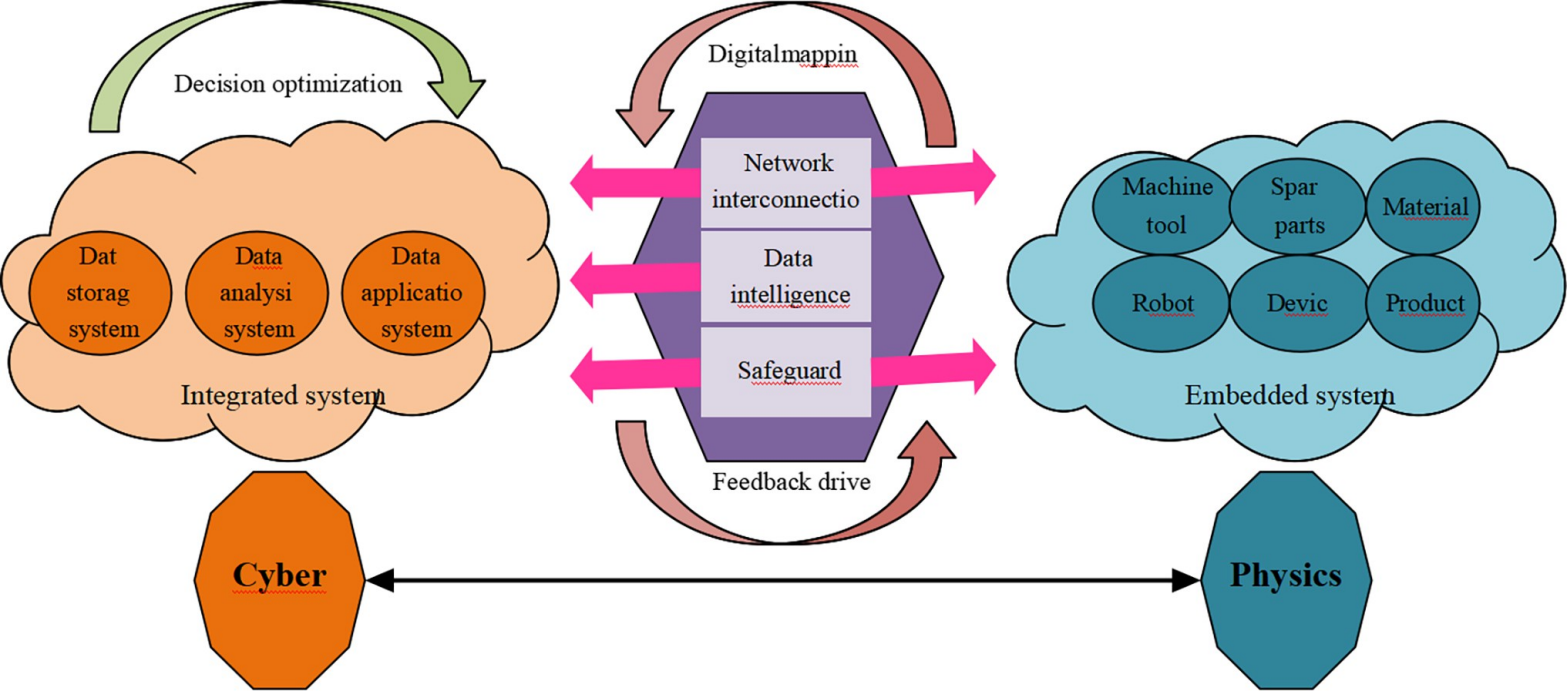
Schematic diagram of CPS.

The energy consumption of embedded systems is mainly due to processors, memory, interface network cards, and external circuit systems. The processor’s energy consumption is the most concerning part of the low-energy scheduling field [11]. At present, the progress of IT has strengthened its connection with the outside world, transforming it into a larger and more open CPS and IoT system. CPS is a multi-dimensional complex system integrating computing, network, and physical environment. It realizes real-time perception, dynamic control, and information service of large-scale engineering systems through the organic integration and deep collaboration of computing, communication, and control technologies. It realizes the integrated design of computing, communication, and physical system, which can make the system more real-time collaborative, efficient, and reliable [12]. CPS includes the ubiquitous system engineering of environment perception, embedded computing, network communication, and network control in the future. It enables the physical system to have computing, communication, precise control, remote cooperation, and autonomy. It pays attention to the close combination and coordination of computing resources and physical resources and is mainly used in some intelligent systems such as device interconnection, IoT sensing, smart home, robots, intelligent navigation, etc. [13]. The biggest difference between it and the IoT is that the IoT is best at wireless connection and mainly realizes perception with little control. In addition to information transmission, CPS has stronger coordination and computing capabilities, thus realizing autonomy. CPS interacts with physical processes primarily through human-computer interaction interfaces. It uses reliable, secure, and remote real-time access to manipulate a physical entity through networked space. Thus, it can be used in the field of film art and dance creation [14]. The details are expressed in Fig 1.

Fig 1 signifies that the system includes system engineering, such as environment perception, embedded computing, network communication, and network control. Therefore, the physical system has functions such as computing, communication, precise control, remote collaboration, and autonomy [15]. The most prominent feature is that it focuses on the tight combination and coordination between computing and physical resources to apply to the interconnection of devices on most intelligent systems. Common electronic devices involve smart grids, industrial control, and robotics [16]. Massive computing is a common feature of CPS access devices, so its access devices generally have mighty computing power. Thereby, from the viewpoint of computing performance, the IoT can be considered a minimalist application of CPS [17].

### Validation of functional safety requirements

**Fig 2:**
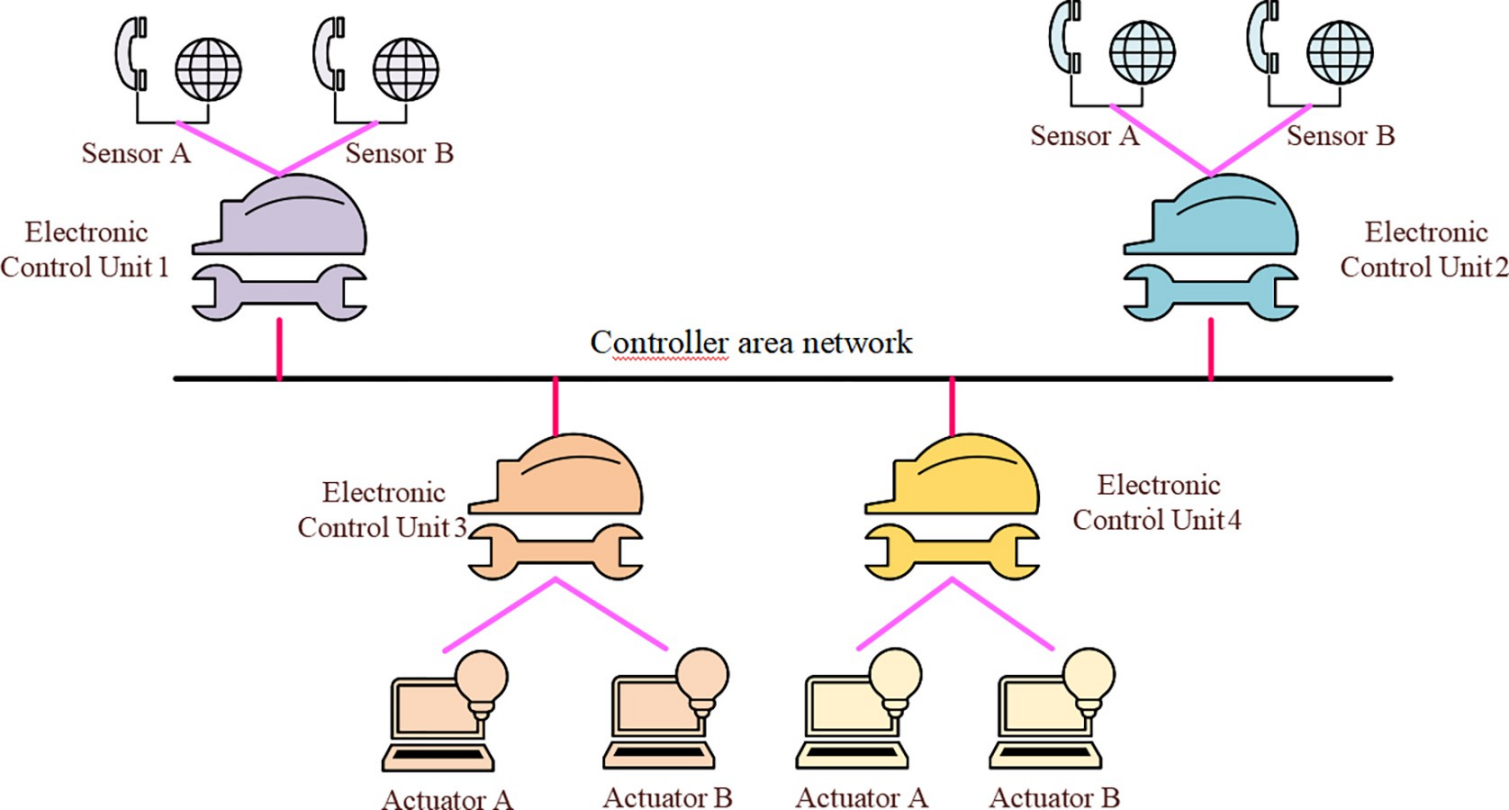
Schematic diagram of common CPS architecture.

Currently, the main challenges in applying CPS to the creative practice of film dance are the system’s real-time, robustness, safety, and energy consumption. It is necessary to solve the problems of functional safety requirement verification and energy consumption optimization under functional safety to implement a highly stable and energy-efficient scheduling algorithm in CPS. Functional safety requirements verification is primarily for transient and permanent hardware failures [18]. Transient failure is a kind of runtime fault, such as a Microsoft error. Permanent failures refer to faults that have occurred and will remain, such as disconnection or lack of connection [19]. The common CPS architecture is outlined in Fig 2.

**Fig 3:**
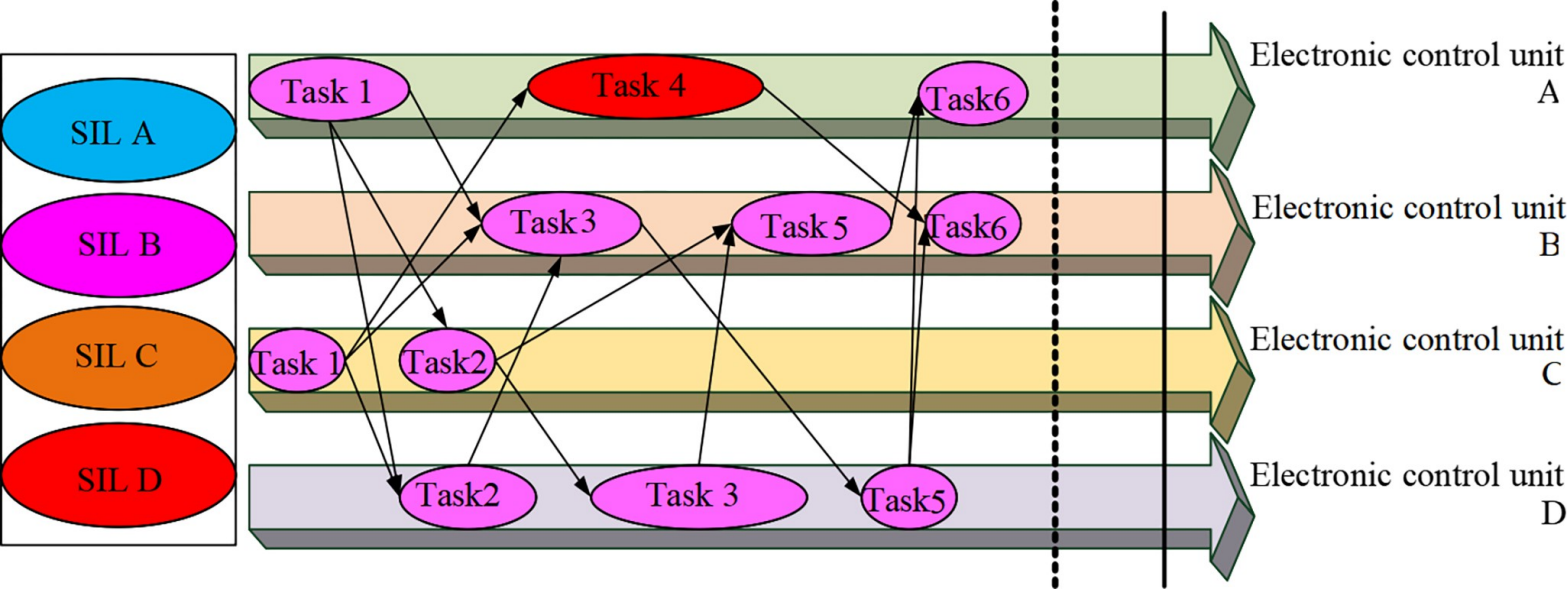
Example of task assignment resulting from application response time requirement verification.

In Fig 2, the control local area network bus is a mediated arbitrated bus triggered by lossless events. It is connected to multiple electronic control units, connecting various sensors and actuators. The application end-to-end calculation and communication process is the feedback cycle process from the sensor to the actuator. Each application task in different electronic control units will send a message to all subsequent tasks after execution in the current electronic control unit [20]. The CPS functional safety critical level is expressed by Safety Integrity Level (SIL) A, SIL B, SIL C, and SIL D. The first step here is to improve application reliability. It ensures the response time requirements of the application. After obtaining the new response time requirements in the response time requirement calculation, the reliability value of each SIL decomposition scheme of the task is calculated [21]. Here, the gap between the response time requirements of the application and the response time to meet the egress task is used to recover this excess time, thereby inversely enhancing the reliability of the application. An example of response time requirement verification is signified in Fig 3.

**Fig 4:**
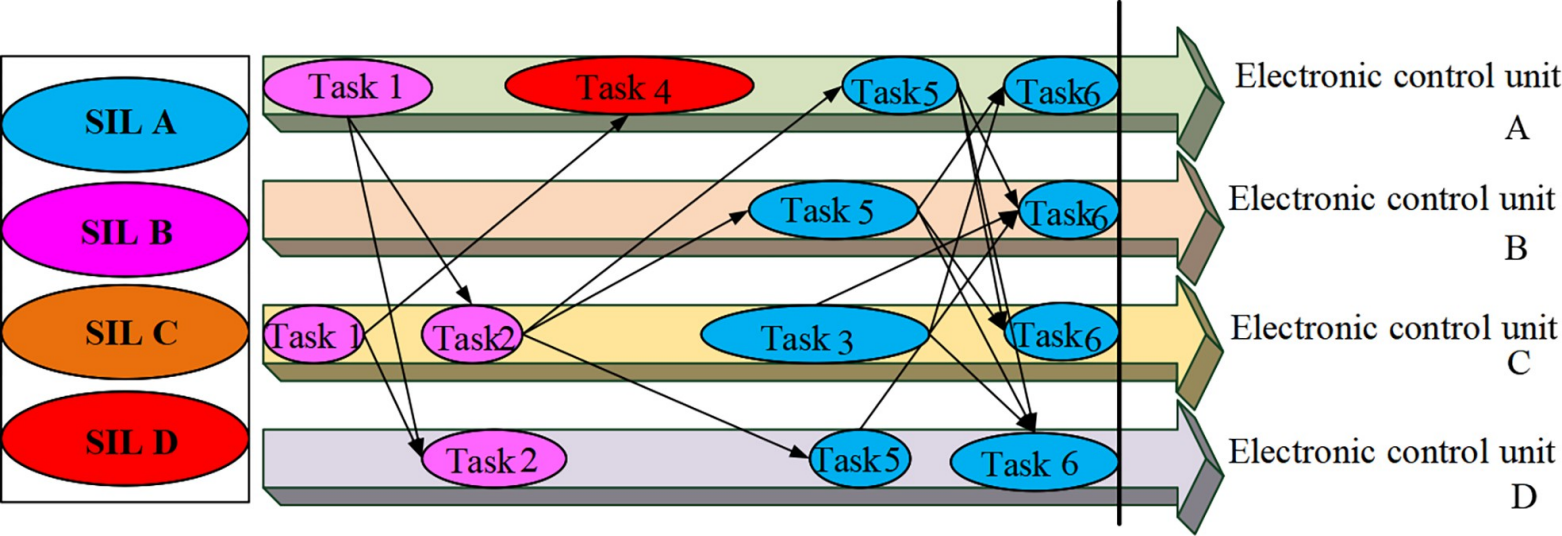
Example of task reassignment resulting from application response time requirement validation.

Fig 3 exhibits that in this example, Task_1_, Task_2_, Task_3_, Task_4_, Task_5_, and Task_6_ represent the tasks of the application. The dashed line on the right represents the resulting response time of t_1_. The solid line on the right represents the response time requirement of the application with t_2_. When the response time of the egress task is less than or equal to the response time requirement of the application, the entire end-to-end node from the egress task to the ingress task can be redistributed, thereby maximizing the reliability of the application [22], as demonstrated in Fig 4.

As outlined in Fig 4, the time complexity is reduced by translating the application’s response time requirements into each task’s response time requirements. Pre-allocation technology ensures the response time requirements of the application. The figure denotes the reassignment process for tasks n_6_ and n_5_. If the actual reliability value obtained is greater than or equal to the reliability requirement when the reliability requirement provided to the application is known, the functional safety requirement verification is satisfied. Otherwise, it will not be passed, and the process will be closed. In this way, the application’s response time requirements are converted to each task’s response time requirements. The SIL decomposition scheme with the largest reliability value is selected in multiple schemes for each task, thereby enhancing the reliability of the application and ensuring its functional safety requirements [23].

### Optimization of system energy consumption under the satisfaction of functional safety requirements

**Fig 5:**
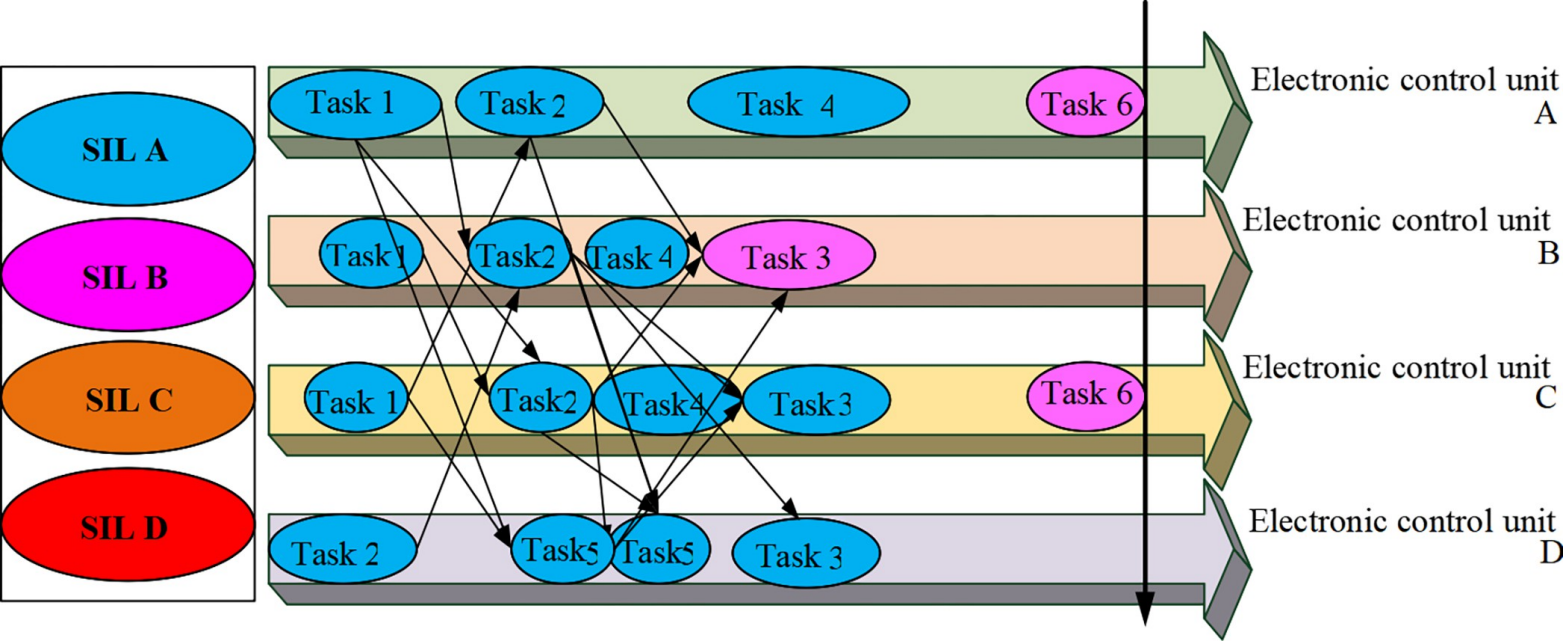
Example of an application of functional safety-critical energy optimization reallocation tasks.

The pre-allocation technology ensures the SIL decomposition scheme for all tasks in the current application. Maximum reliability can be achieved under the premise of ensuring the response time requirements of the application. Based on this, the system energy consumption is optimized. The main way is to optimize the SIL scheme for each task in the pre-allocation phase, thereby reducing the energy consumption of the system application [24]. The specific operation steps are as follows. First, the system-level power model and list scheduling are employed to transfer the application’s functional safety requirements to each task’s functional safety requirements. Then, the current task’s response time and reliability requirements are determined. Next, the energy consumption is calculated. Ultimately, the SIL decomposition scheme with the lowest energy consumption for this task is selected [25]. The specific reallocation process is revealed in Fig 5.

Fig 5 denotes that the main idea is to shift the application’s response time and reliability requirements to each task’s response time and reliability requirements. Task n_5_ in the figure determines the current task’s response time and reliability requirements and assigns the various solutions obtained. The final practical application’s reliability, response time, and energy consumption are calculated. Next, the priority sequence is performed. Finally, the lowest energy consumption of all SIL decomposition schemes is chosen to meet functional safety requirements [26]. The rest of the tasks are the same as n_5_, and the SIL decomposition scheme with the lowest energy consumption is selected.

### Construction of low-energy parallel scheduling algorithm

**Fig 6:**
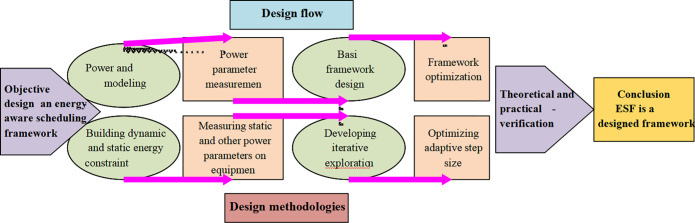
Flowchart of EAS framework design.

After solving the above problems, the problem of low-energy parallel scheduling in the filming equipment for film and dance creation is analyzed. The essence is to optimize the response time of the joint constraint of dynamic and static energy under uncontrollable static energy consumption [27]. Therefore, a new EAS framework is proposed to ensure dynamic and static energy constraints. It adopts the existing energy pre-allocation technology as the scheduling component in the framework. The specific process is suggested in Fig 6.

**Fig 7:**
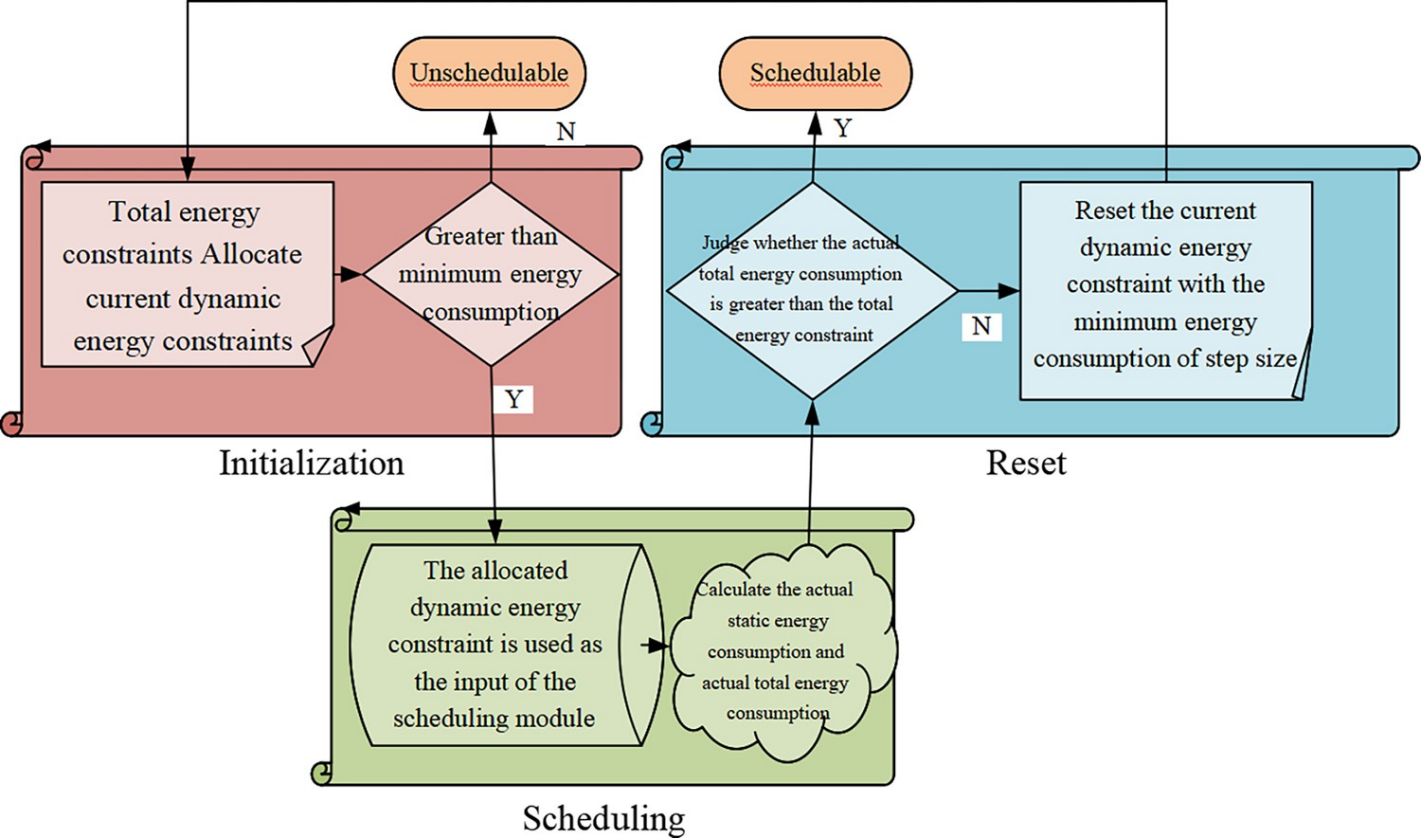
Basic framework design flowchart.

The new EAS framework process is divided into four parts in Fig 6. Power and energy modeling refers to establishing hybrid energy constraint models that include dynamic and static energy constraints. Power parameter measurement is the measurement of real industrial equipment’s static power and other power parameters. The design of basic framework is completing basic framework construction through iterative exploration. Framework optimization is to perform adaptive step selection to optimize the basic framework [28]. The energy pre-allocation technique is adopted to schedule the internal components of the new framework so that the existing energy pre-allocation technology can ensure the constraints of the actual combination of dynamic and static. The detailed design of the basic framework is presented in Fig 7.

Fig 7 illustrates that the framework design mainly consists of three stages: initialization, scheduling, and reset [29]. The initialization stage assigns the total energy constraint to the current dynamic energy constraint. In the scheduling stage, the allocated dynamic energy constraint is used as the input of the scheduling module to obtain the actual dynamic energy consumption and response time and calculate the actual static energy consumption and the total energy consumption [30]. In the final reset phase, whether the actual total energy consumption is greater than the total energy constraint is judged first. If it exceeds the total energy constraint, the current dynamic energy constraint is reset with the step minimum energy consumption to return to the initialization stage again. Finally, a three-stage loop is made until the framework returns schedulable or non-schedulable [31]. The basic framework guarantees dynamic and static energy constraints simultaneously, but time performance is poor. Thus, static and dynamic step sizes should be selected experimentally. The static step size does not change with each iteration. However, the dynamic step size can be adaptively updated in each iteration, which is the actual static energy consumption in the current iteration [32]. The application and actual energy constraints are input to each scheduling component of the ESF to input all steps to the reset stage of the basic framework. Each scheduling component’s optimal step size is obtained by calling multiple basic framework instances in parallel. The above process is repeated a specified number of times, and the count of all output steps is recorded. The higher the number of step outputs, the better the schedulability of the base framework at this step [33].

## Results and discussion

The operating system used in this study is Windows10 64-bit system, the Central processing unit (CPU) is Intel(R)Core(TM)i7-7700, the CPU frequency is 3.60GHz, the memory is 16GB, and the core is 16GB. The graphics card is NVIDIA GeForce GTX 1080 x 4, and the storage disk is 5.2TB with 8GB of video memory.

### Schedulability experiment results and analysis

**Fig 8:**
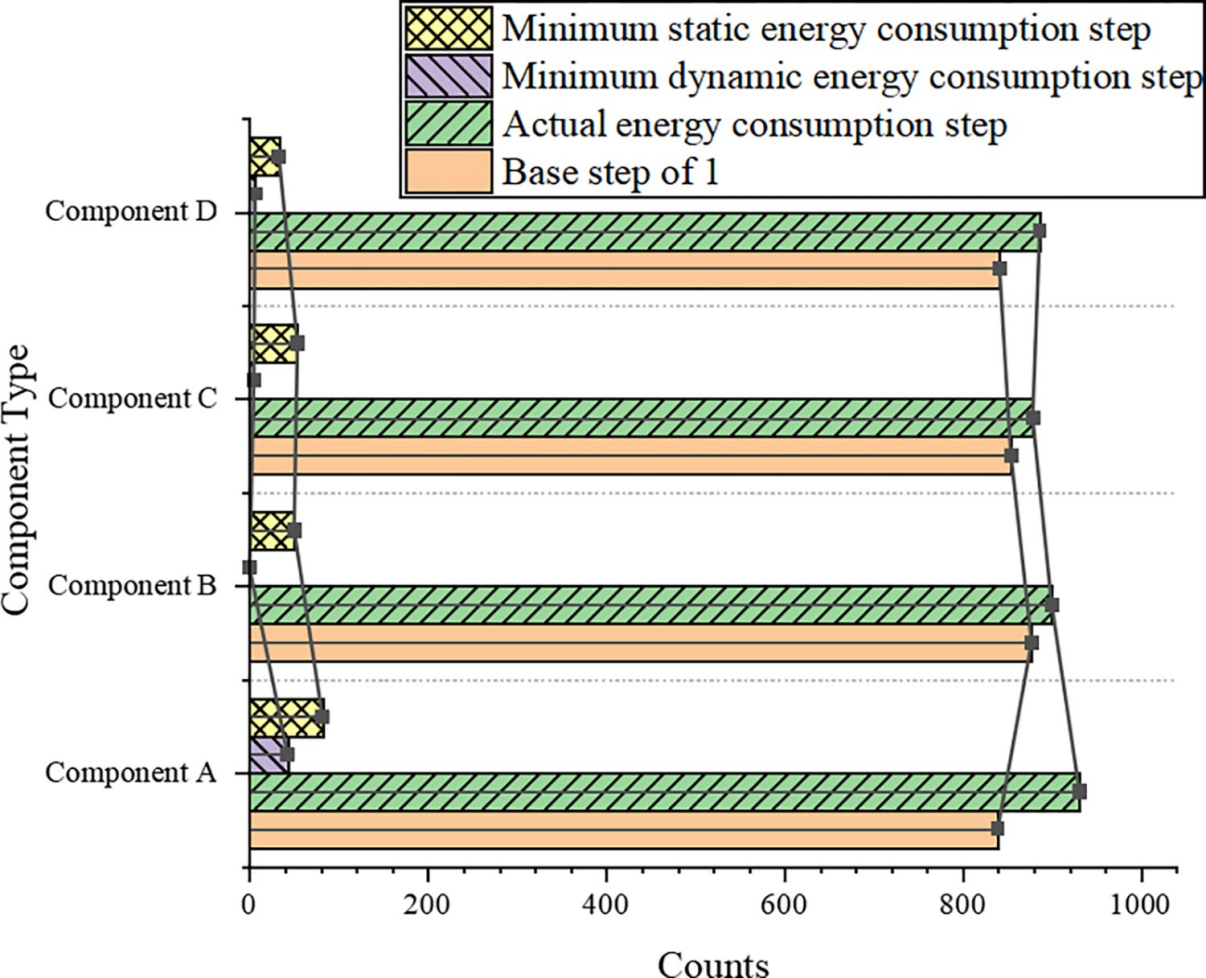
Instance count of all output steps based on schedulability.

Here, the schedulability and time performance of the basic framework are used as the selection criteria for the optimal step size of each scheduling component. The Fast Fourier Transform (FFT) and Gaussian elimination structure are applied. The instance count of all output steps based on schedulability is demonstrated in Fig 8.

**Fig 9:**
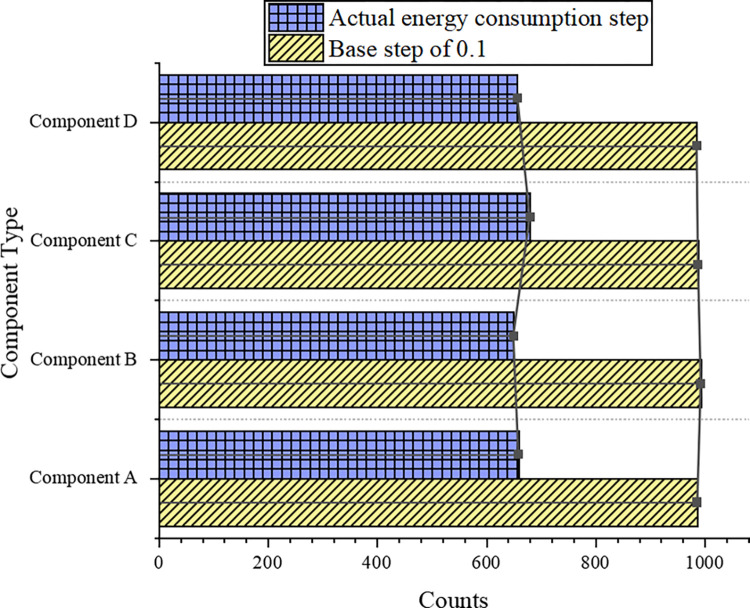
Comparison of step size 0.1 with the actual energy consumption step count.

In Fig 8, the FFT structure results present that the counts of the minimum dynamic energy step and the minimum static energy step are much smaller than the actual static energy step and 1 in the current iteration. The minimum dynamic energy consumption step count is almost 0. The actual static energy step in the current iteration can get the most count. In 1000 experiments for the four scheduling components, this step obtained an average of at least 900 counts or more. Thus, in an FFT structure, the actual static step size in the current iteration allows for the best schedulability. Step 1 is actually an iteration of dynamic energy constraints by sacrificing brute force search for time complexity, so it should get the maximum output count in 1,000 experiments. However, Fig 8 describes that the actual energy consumption step count in the current iteration exceeds the baseline step 1. Consequently, the baseline step size is changed from 1 to 0.1. The experiment is re-run. The comparison results are plotted in Fig 9.

Fig 9 portrays that for each scheduling component, the output count with a step of 0.1 obtains a larger output count than the actual energy consumption step. Thereby, the schedulability of the actual energy consumption step is better than that of step 1. The average number of iterations analyzed under 1,000 experiments shows that a basic framework instance with a step size of 0.1 requires an average of nearly 800 iterations to obtain a schedulable solution. The basic framework instance of the actual energy consumption step only needs 7–8 iterations, which indicates that the dynamic step size can enable the basic framework to obtain better schedulability with lower time complexity.

### Time performance experimental results and analysis

**Fig 10:**
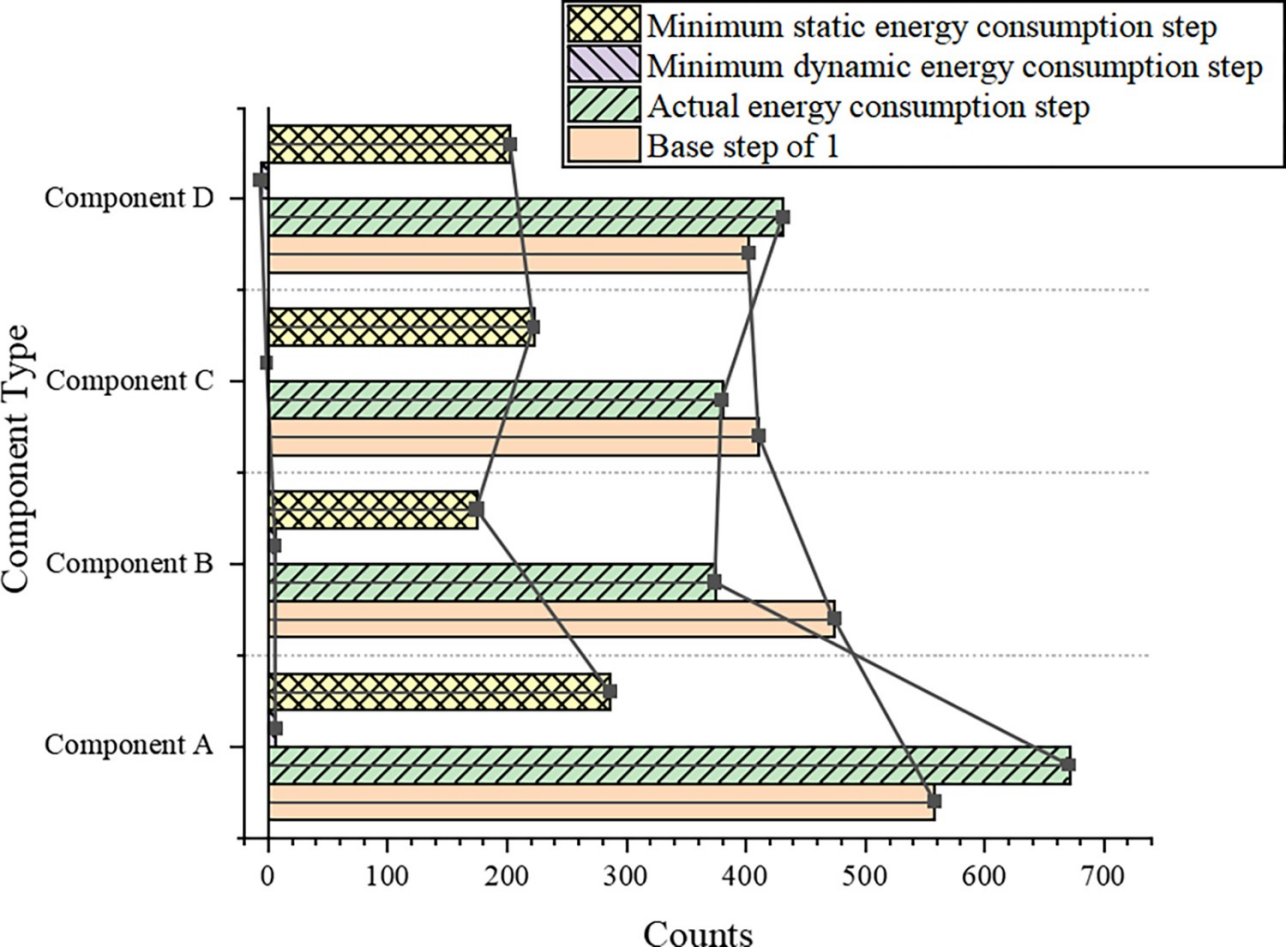
Count comparison of all output steps based on time performance.

Experiments and analyses of time performance are performed. The total energy constraint is set to a fixed 0.5× actual total energy consumption in all 1,000 experiments. The step size of a basic framework instance that achieves the minimum response time is output. After 1000 repeats, the counting results for all output steps are recorded, as illustrated in Fig 10.

Fig 10 displays that in parallel applications of FFT structures, the minimum dynamic energy step and the minimum static energy step count are still the least. The minimum dynamic energy step is counted at zero, indicating that none yielded an optimal response time value. The counts for dynamic step size and baseline step 1 are still close. The actual energy consumption step from the various components is actually an adaptive step size, which can reasonably reflect the dynamic and static energy constraint ratio. As a result, the basic framework achieves better time performance than other step sizes. Moreover, the actual energy consumption step is selected as the step size of the reset stage for the basic framework.

### Theoretical energy consumption evaluation on a simulation platform

**Fig 11:**
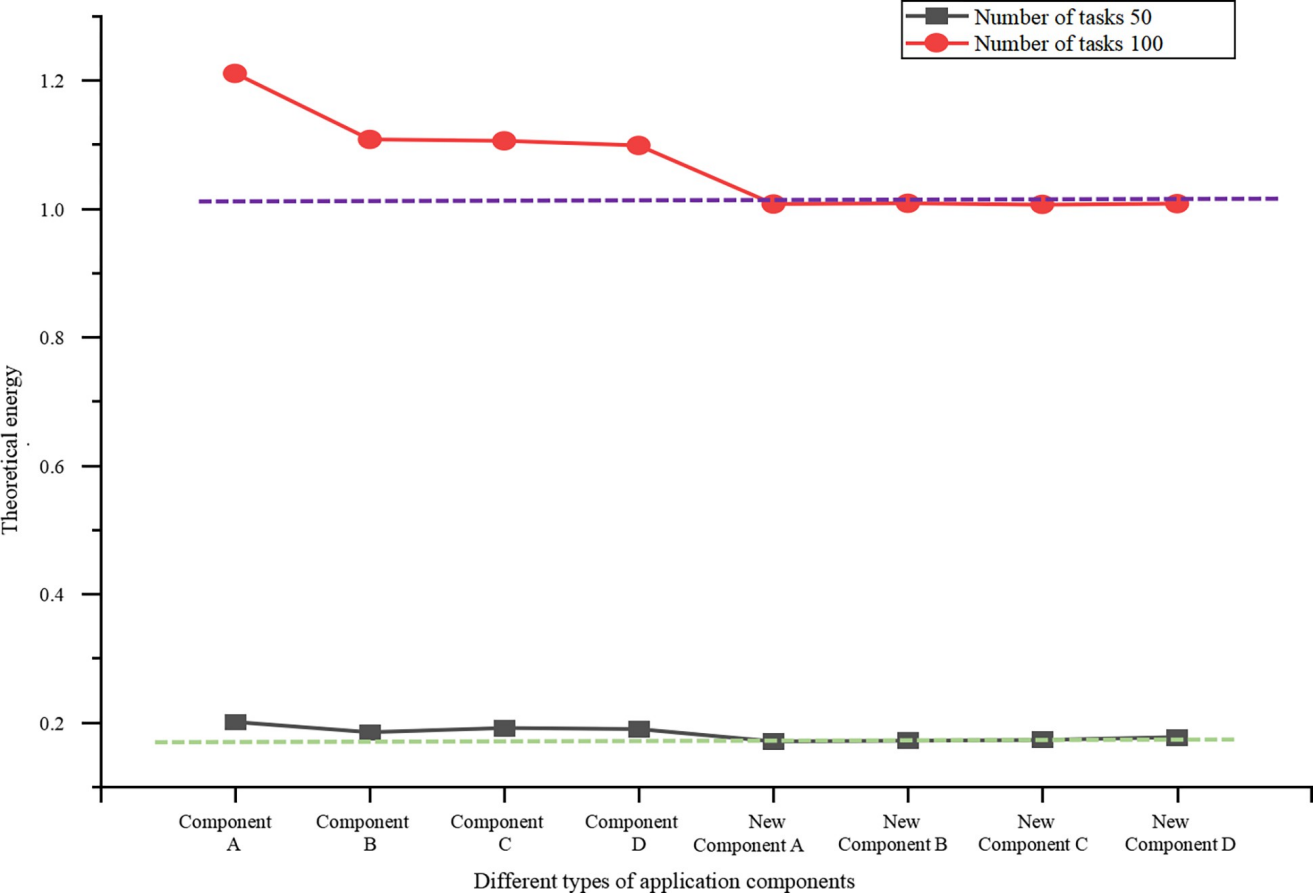
Theoretical energy consumption on a simulated platform.

The theoretical energy consumption analysis is carried out on the simulation platform. The theoretical energy consumption results of the application of the FFT structure under the change of task number are shown in Fig 11.

The two dashed lines in Fig 11represent the actual energy constraints for diverse numbers of tasks. It is found that all energy pre-allocation technologies consume more than the actual energy limit. The corresponding new basic framework can ensure that the actual energy consumption is within the actual energy constraints, indicating that both dynamic and static energy constraints can be satisfied. The energy consumption of all scheduling components is reduced and maintained within a specific range. Consequently, it is explained that under the condition of the simulation platform, the newly constructed basic framework can be applied to energy sensing equipment to a certain extent and can solve the problem of energy sensing scheduling under the constraint of ensuring dynamic and static energy.

## Discussion

In conclusion, the proposed film creation scheduling algorithm based on CPS with high stability and energy efficiency has better time performance and energy consumption constraints. Additionally, it was compared with other similar studies. Qin et al. (2020) proposed an energy-aware multi-objective reinforcement learning (RL) algorithm based on the RL of the Chebyshev function, and compared the proposed algorithm with two state-of-the-art multi-objective meta-heuristic methods for four different workflows. The results indicated that their proposed algorithm was superior to these existing methods [34]. Compared with this study, both of them proposed an EAS algorithm, but the methods used were different. They used the Chebyshev function, while CPS was used here. Lu et al. (2022) put forward an iterative greedy tabu mechanism algorithm for scheduling in dynamic mobile cloud computing environments, involving tradeoff decisions of power consumption, deadline, and system load. Compared with recent methods such as adaptive first-come, first-served, minimization of execution time, and code offloading tradeoff decisions, simulation results show that their proposed algorithm reduces energy consumption and increases the number of completed jobs [35]. Their research was mainly applied to mobile cloud computing, and compared with this study, both reduced energy consumption, but the algorithm proposed here also reduced time. Shabestari et al. (2022) proposed a deadline aware scheduling algorithm based on the moth flame optimization (MFO) to minimize energy consumption and execute the application within a given soft deadline. The results manifested that the proposed method outperformed the energy-aware greedy algorithm and the cut-time-aware energy-efficient MapReduce scheduling algorithm in terms of total cluster energy consumption and meeting the operation period [36]. Both the method and this study reduced energy consumption while ensuring the quality of work.

## Conclusions

Innovation is the core driving force of social development. Cross-border cooperation in artistic innovation is the most common way of creative innovation. This study addresses the conflicting problem of high performance and energy awareness of equipment in film and dance creation. Firstly, the high-efficiency functional safety issues in the CPS are explored. Secondly, the verification analysis of functional safety requirements and the optimization of energy consumption to meet functional safety requirements are carried out. Finally, a low-energy parallel scheduling algorithm for industrial equipment is proposed. A four-stage integrated process and design ensure that its basic framework guarantees dynamic and static energy constraints for parallel applications. From the experimental results of the framework optimization and simulation platform, the scheduling algorithm has advantages in optimizing energy consumption. It can shorten the development life cycle of CPS parallel applications. From an industrial point of view, the proposed energy-efficient functional safety scheduling algorithm has practical significance for the energy-sensitive and environmentally-friendly film industry. However, this study only conducts experimental analysis on the simulation platform and does not apply the proposed algorithm to film equipment. Moreover, the analysis of the algorithm is not deep enough. Therefore, in the future, it is necessary to deeply combine the research results with the actual equipment system with a complex hardware environment and customize appropriate optimization algorithms according to each hardware feature. Besides, advanced algorithms such as machine learning should be introduced to break through the optimization bottleneck of the CPS. Thus, the advantages of film and dance can be combined to create works of art that conform to the aesthetics of modern society.
